# Editorial: Ecology, Virulence, and Detection of Pathogenic and Pandemic *Vibrio parahaemolyticus*

**DOI:** 10.3389/fmicb.2016.00156

**Published:** 2016-02-16

**Authors:** Iddya Karunasagar, Indrani Karunasagar, Pendru Raghunath

**Affiliations:** ^1^Nitte University Center for Science Education and Research (NUCSER), Nitte UniversityMangalore, India; ^2^Department of Microbiology, School of Medicine, Texila American UniversityGeorgetown, Guyana

**Keywords:** *Vibrio parahaemolyticus*, pathogen, marine, seafood, virulence, ecology, pandemic, detection

*Vibrio parahaemolyticus* is a very versatile halophilic organism that can adapt to a wide variety of environments and can cause infections in both humans and aquatic animals. This versatility in terms of host and habitat is attributable to the ability to acquire genes that improve fitness of the organism in different situations. Several genomic islands have been described in this organism. Human pathogenic strains are characterized by the presence of pathogenicity islands that encode certain specific Type Three Secretion Systems (TTSS) and hemolysins that are not present in most environmental strains (Chen et al., 2011). Recently characterized shrimp pathogenic *V. parahaemolyticus* strains have plasmid borne virulence genes (Sirikharin et al., 2015). *V. parahaemolyticus* is associated with zooplankton like copepods in off-shore waters and same genotype has been found over large areas (Martinez-Urtaza et al., 2012). The global spread of pandemic *V. parahaemolyticus* has been attributed to the El Nino phenomenon characterized by the arrival of equatorial warm waters to South American coast in a sequence of invasive waves lasting about 6 months in 1997 (Martinez-Urtaza et al., 2008). Studies conducted using molecular techniques such as realtime PCR and multilocus sequence typing have helped detecting pathogenic *V. parahaemolyticus* in environmental samples and in understanding their global spread.

Thus, this organism has attracted attention of both seafood safety managers as well as aquatic animal health professionals. Being an autochthonous aquatic organism, *V. parahaemolyticus* has global distribution, occurring wherever environmental conditions are favorable. *V. parahaemolyticus* is a model organism for the “one health” concept, which recognizes that human health is connected to the health of animals and the environment. In order to better manage both public health and aquatic animal health, we need a better understanding of the factors effecting the ecology of this organism, the virulence factors present in human and animal pathogenic strains.

The papers in this research topic cover the three major aspects of pathogenic and pandemic *V. parahaemolyticus*: ecology, virulence, and detection. Lopez-Joven et al. discuss the prevalence of pathogenic and non-pathogenic strains in association with molluscs while Zavala-Norzagaray et al. describe *Vibrio* spp. associated with sea turtles in Mexico. Host colonization depends on the ability of the organism to acquire difficult to get nutrients such as iron. León-Sicairos et al. describe strategies of *V. parahaemolyticus* to obtain iron. Improvements in the detection methods of pathogenic strains has been presented by Escalante-Maldonado et al. Genetic characterization of clinical and environmental strains has enabled Xu et al. to understand the emergence of indigenous and non-indegenous pathogen lineages. Genomic and molecular typing studies provide insights into the environmental reservoirs and genetic diversity of pathogenic and pandemic strains as described by Hazen et al., de Jesús Hernández-Díaz et al., Lüdeke et al., and Haendiges et al. Function of genes involved in Type IV secretion system of *V. parahemolyticus* has been investigated by Yu et al. and conditions leading to loss of plasmid in this organism has been described by Letchumanan et al. Raghunath presented insights into the role of virulence genes involved in human infections. Thus, the articles presented in this research topic contribute to a better understanding of the ecology, virulence, and detection of this important aquatic organism that impacts for both public health and aquaculture.

## Author contributions

All authors listed, have made substantial, direct, and intellectual contribution to the work, and approved it for publication.

### Conflict of interest statement

The authors declare that the research was conducted in the absence of any commercial or financial relationships that could be construed as a potential conflict of interest.
